# 78 Assessment of the Scalpel Blade as a Calibration Tool for Measuring Dermatome Cut Thickness

**DOI:** 10.1093/jbcr/irae036.077

**Published:** 2024-04-17

**Authors:** Dallan Dargan, Sebastian Q Vrouwe, Lawrence Gottlieb

**Affiliations:** University of Chicago Medicine Center, Chicago, Illinois; University of Chicago, Chicago, IL; University of Chicago Medicine Center, Chicago, Illinois; University of Chicago, Chicago, IL; University of Chicago Medicine Center, Chicago, Illinois; University of Chicago, Chicago, IL

## Abstract

**Introduction:**

Dermatomes are widely used for split thickness skin graft harvest and are regarded as the optimal means of obtaining a graft of the desired thickness. The harvested graft thickness depends in part on the cut thickness setting, adjusted using a dial on the dermatome, according to increments in thousandths of an inch, or millimetres. Previous authors have described a simple “on table” technique which uses a standard scalpel blade as a calibration tool to determine cut thickness immediately prior to graft harvest. This study aims to assess how reliable and consistent the dial on the dermatome is, and whether a #15 surgical blade placed in the dermatome blade aperture may be used to set or confirm the cut thickness. In addition, whether any statistical or clinical difference exists between the blade-measured aperture and the stated aperture using either the cutting (belly) or non-cutting edge (spine) of the scalpel, as well as the intra- and inter-rater reliability of the technique.

**Methods:**

The precise thickness of #15 surgical blades (N=10) was first measured using an electronic micrometer. Six working dermatomes, loaded with a four inch guard, were each assessed by two surgeons in triplicate using both the belly and spine of the blade. The endpoint was the smallest cut thickness setting that would permit passage of an entire surgical blade through the dermatome aperture, starting from zero and increasing cut thickness at intervals of 1/1000 in.

**Results:**

The mean measured #15 scalpel blade thickness was 0.391 mm (range 0.381-0.408 mm), or 16/1000 in. The mean cut thickness setting, of all 72 measurements, which would permit the blade was 6.0/1000 in (SD = 1.6/1000 in). This was a mean difference of 10/1000 in from the measured thickness of a #15 scalpel blade, one-way ANOVA p< 0.001. Individual dermatome apertures differed, ranging from a mean of 5.0 to 7.8/1000 in, ANOVA p< 0.001. Intra-rater reliability of this technique was excellent (ICC = 0.89), however inter-rater reliability was only fair (ICC = 0.52), with a mean difference of 1.5/1000 between surgeons (6.8 vs 5.2/1000 in, t-test p< 0.018). The mean difference between passing the belly versus the spine of the blade was not significant, 0.47/1000 in, t-test p=0.474.

**Conclusions:**

Testing the aperture opening with a #15 blade is a more consistent measurement of thickness then reading the number on the dial of the dermatome. A clinically significant difference exists between the thickness of a #15 surgical blade and the stated cut thickness of a dermatome. This ad hoc calibration technique has only modest reliability between individuals, however the difference in thickness may not be clinically significant. Using the belly or spine of the scalpel produces similar results.

**Applicability of Research to Practice:**

The test is a simple and reproducible manoeuvre to calibrate a dermatome, however there appears to be a large discrepancy between the stated cut thickness and physical dimensions of a scalpel blade.

